# Viral genotype correlates with distinct liver gene transcription signatures in chronic hepatitis C virus infection

**DOI:** 10.1111/liv.12830

**Published:** 2015-04-07

**Authors:** Mark W. Robinson, Elihu Aranday‐Cortes, Derek Gatherer, Rachael Swann, Jolanda M. P. Liefhebber, Ana Da Silva Filipe, Alex Sigruener, Stephen T. Barclay, Peter R. Mills, Arvind H. Patel, John McLauchlan

**Affiliations:** ^1^ MRC – University of Glasgow Centre for Virus ResearchGlasgowUK; ^2^ School of Biochemistry and ImmunologyTrinity College DublinDublinIreland; ^3^ Division of Biomedical and Life SciencesLancaster UniversityLancasterUK; ^4^ Gartnavel General HospitalNHS Greater Glasgow and ClydeGlasgowUK; ^5^ Institute of Clinical Chemistry and Laboratory MedicineRegensburg University Medical CenterRegensburgGermany; ^6^ Glasgow Royal InfirmaryNHS Greater Glasgow and ClydeGlasgowUK

**Keywords:** genotype, HCV, interferon, ISGs, transcriptomics

## Abstract

**Background:**

Chronic hepatitis C virus (HCV) infection of the liver with either genotype 1 or genotype 3 gives rise to distinct pathologies, and the two viral genotypes respond differently to antiviral therapy.

**Methods:**

To understand these clinical differences, we compared gene transcription profiles in liver biopsies from patients infected with either gt1 or gt3, and uninfected controls.

**Results:**

Gt1‐infected biopsies displayed elevated levels of transcripts regulated by type I and type III interferons (IFN), including genes that predict response to IFN‐α therapy. In contrast, genes controlled by IFN‐γ were induced in gt3‐infected biopsies. Moreover, IFN‐γ levels were higher in gt3‐infected biopsies. Analysis of hepatocyte‐derived cell lines confirmed that the genes upregulated in gt3 infection were preferentially induced by IFN‐γ. The transcriptional profile of gt3 infection was unaffected by *IFNL4* polymorphisms, providing a rationale for the reduced predictive power of *IFNL* genotyping in gt3‐infected patients.

**Conclusions:**

The interactions between HCV genotypes 1 and 3 and hepatocytes are distinct. These unique interactions provide avenues to explore the biological mechanisms that drive viral genotype‐specific differences in disease progression and treatment response. A greater understanding of the distinct host–pathogen interactions of the different HCV genotypes is required to facilitate optimal management of HCV infection.

**Abbreviations**
DAAs: direct‐acting antivirals
gt1: genotype 1
gt3: genotype 3
HCV: hepatitis C virus
IFN: interferon
ISG(s): interferon‐stimulated gene(s)
RIN: RNA integrity number
RNA‐Seq: RNA sequencing
SVR: sustained virological response
Q‐PCR: quantitative PCR

**Key Points**

- Transcriptional profiling of liver tissue from hepatitis C virus‐infected individuals can identify distinct viral genotype‐specific host–pathogen interactions.
- Genotype 1 infection is associated with an elevated type I IFN‐like response and is influenced by host *IFNL4* genotype.
- Genotype 3 infection is associated with transcription of genes involved in cell‐mediated immune responses and IFN‐γ signalling, independent of host *IFNL4* genotype.
- Understanding the influence of viral genotype on hepatitis C disease progression will facilitate the optimal clinical management of different hepatitis C virus genotypes, the development genotype‐specific predictors of therapy and the efficient utilisation of antiviral therapeutics.

Hepatitis C virus (HCV) is a highly diverse human pathogen, which is classified into seven distinct genotypes that vary by >30% at the nucleotide sequence level 1. The virus establishes a chronic infection within liver hepatocytes and is one of the leading causes of liver disease worldwide 2. The development of novel direct‐acting antivirals (DAAs) with higher efficacy than interferon (IFN) treatment 3, 4, and the first prophylactic vaccines moving into human trials 5, raises the possibility of combating HCV infection on a global scale. However, presently licensed DAAs act on a limited range of viral genotypes and the vaccines under development target only genotype (gt)1 viral antigens. While gt1 is prevalent worldwide (46.2% of HCV cases), over half of all infections are caused by other genotypes 6. Among these other HCV genotypes, gt3 is the most prevalent (accounting for 54.3 million cases, 30.1%) 6. Achieving HCV eradication requires a better understanding of the host–pathogen interactions that influence the natural history of each viral genotype.

Viral genotype is an important clinical indicator in the management of HCV infection, affecting both disease progression and treatment options. Gt3 infection is associated with decreased peripheral cholesterol levels and an increased prevalence of liver steatosis, which resolve upon successful viral clearance 7. There is also evidence that gt3 infection is independently associated with faster liver fibrosis progression 8. From the perspective of antiviral therapy, gt1‐infected patients show a reduced response to interferon‐based regimens (40–50% sustained virological response [SVR] for gt1 compared to 70–80% for gt3) 9. Conversely, phase 3 trials of pan‐genotypic DAAs indicate gt3‐infected patients are more difficult to treat with IFN‐free therapies 3.

Microarray analysis of hepatic gene expression has yielded valuable information on host–pathogen interactions during chronic HCV infection. From initial studies in chimpanzees, persistently infected animals displayed elevated transcription of genes including the following: (i) antiviral interferon‐stimulated genes (ISGs), (ii) modulators of immune cell activity and (iii) regulators of lipid metabolism 10, 11. The same approach for analysis of human liver biopsies has identified similarities in the patterns of gene expression during chronic infection 12, 13, 14, 15. These human and chimpanzee studies have largely focused on gt1 infection with limited data analysis on other viral genotypes, including gt3.

In this report, we set out to explore distinct host responses induced by different HCV genotypes. We hypothesise that the observed clinical features of gt1 and gt3 infection are related to fundamental differences in the nature of the virus–host interactions within liver cells. To explore this hypothesis, we have analysed the transcriptional changes in infected liver tissue using high‐throughput RNA sequencing (RNA‐Seq). This technology offers a broader dynamic range than microarray analysis and enables analysis of novel unannotated RNA transcripts. Our study aimed to create a transcriptional profile of gt1 and gt3 infection, as a platform to understand factors influencing the genotype‐specific progression of liver disease and the response to antiviral therapy.

## Materials and methods

### Clinical samples

Clinical samples were used with informed consent, conforming to the ethical guidelines of the 1975 Declaration of Helsinki. Study protocols were approved by the West of Scotland Research Ethics Committee and the Tayside Tissue Bank Access Committee. Liver needle biopsies from patients with chronic HCV infection (gt1, *n *=* *9; gt3, *n *=* *9), carried out as part of routine clinical care, were divided, with half fixed for routine histological examination and the remaining half snap‐frozen in liquid nitrogen. Seven of the HCV‐infected individuals had previously failed non‐pegylated interferon therapy (4 gt1 and 3 gt3; Table 1), but none were receiving interferon therapy at the time of liver biopsy.

**Table 1:** Clinical data for liver biopsies

	Genotype 1	Genotype 3	Control
*n*	9	9	6
Male:Female	5:4	6:3	2:1^a^
Previous Treatment Failure (Y:N)	4:5	3:6	n/a
rs368234815 Genotype (*TT/TT*:*TT/ΔG*:*ΔG/ΔG*)^b^	2:5:1	6:3:0	3:2:1
Ishak Inflammation Score (1‐18)	3 (2‐5)	4 (2‐7)	n/a
Ishak Fibrosis Score (0‐6)	0 (0‐3)	0 (0‐6)	n/a
METAVIR Fibrosis Score (0‐4)	0 (0‐2)	0 (0‐4)	0.5 (0‐1)

n/a, not applicable.

All values are given as the median with the range in brackets.

a Gender was unavailable for 3 anonymised control samples.

b rs368234815 genotype was unavailable for one gt1‐infected patient.

As a control group, RNA was extracted from uninfected liver biopsy and resection samples (*n *=* *6). These included liver samples from patients with conditions that are associated with hepatic disease (one each with genetic haemochromatosis, non‐alcoholic steatohepatitis and psoriasis) as well as three normal samples obtained during liver resection for metastatic colorectal adenocarcinoma. Clinical data are detailed in Table 1. Q‐PCR analysis of conserved sequences in the 5′ untranslated region of the viral genome 16 confirmed that only liver biopsies from infected patients had detectable HCV RNA, and no difference in intrahepatic viral RNA levels was found between either gt1‐ or gt3‐infected patients (data not shown).

### RNA extraction

Snap‐frozen liver biopsies were homogenised in 3 ml buffer RLT (Qiagen, Manchester, UK), supplemented with 30 μl β‐mercaptoethanol, using a hand‐held power homogenizer (Polytron; Kinematica AG, Lucerne, Switzerland), and total RNA was extracted using RNeasy Minicolumns with on‐column DNase digestion (Qiagen). RNA quality and quantity were assessed on a 2200 TapeStation (Agilent Technologies, Santa Clara, CA, USA).

### Illumina RNA‐Seq

Samples were prepared with the Illumina TruSeq RNA sample preparation kit (Illumina, San Diego, CA, USA) and sequenced on an Illumina Genome Analyzer IIx (Glasgow Polyomics, University of Glasgow, Glasgow, UK). Raw FASTQ sequencing files (73 base reads) were quality‐filtered for low‐quality bases and adaptor sequences with Trim Galore! v0.2.4 (http://www.bioinformatics.bbsrc.ac.uk/projects/trim_galore/). The RNA‐Seq analysis was performed using the Tuxedo protocol 17, with full details provided in the supplementary methods. Differential gene expression was considered significant when the observed fold change of reads (∆FPKM(log2)) was ≥1.0 and FDR/q‐value <0.05 between comparisons. Pathway analysis was carried out using IPA (Ingenuity Systems, Redwood City, CA, USA).

### Quantitative real‐time PCR (Q‐PCR) and genotyping

Total RNA was reverse‐transcribed with the SuperScript^®^ VILO^™^ Kit (Life Technologies, Paisley, UK), and gene expression was measured by quantitative real‐time PCR (Q‐PCR) using TaqMan^®^ assays (Life Technologies; Table S1). Statistical significance was calculated using the Kruskal–Wallis test in GraphPad Prism 5 (GraphPad Software, La Jolla, CA, USA). Genotyping of *IFNL4* rs368234815 was performed on cDNA using the assay described previously 18 with Type‐it Fast SNP Probe PCR Master Mix (Qiagen).

### Microarray analysis of IFN‐α‐ and IFN‐γ‐stimulated hepatocyte‐derived cell lines

Human hepatocyte‐derived cells (HuH‐7 and HepaRG) were mock‐treated or incubated with 100 IU of IFN‐α (Merck, Kenilworth, NJ, USA) or IFN‐γ (Roche, Basel, Switzerland) for 24 h (*n *=* *2). Total cellular RNA was extracted as above, and 300 ng of RNA was hybridised onto modified Agilent 4 × 44K microarrays containing 201 additional probes, corresponding to 119 genes previously not found on the array (Agilent Technologies). Raw data were extracted using Feature Extraction software (Agilent Technologies) and analysed with ChipInspector (Genomatix Software, Munich, Germany). Fold change values were calculated for each gene in response to IFN‐α and IFN‐γ (mean signal value treatment/mean signal value mock), as well as the ratio of responsiveness to IFN‐α versus IFN‐γ.

## Results

### Viral genotypes induce distinct liver transcriptomic profiles

**Figure 1:**
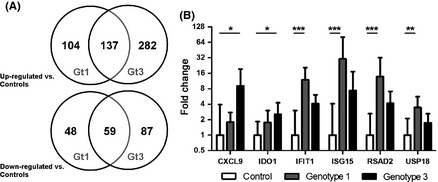
Transcriptional profiles of liver biopsies from HCV gt1‐ and gt3‐infected individuals compared with controls. (A) Significantly upregulated and downregulated genes (∆FPKM(log2) ≥1.0 and q‐value <0.05) in gt1‐ and gt3‐infected patients (each *n *=* *5), versus controls (*n *=* *4). (B) Validation by Q‐PCR (geometric mean with 95% confidence interval) in gt1‐infected patients (*n *=* *9, except *CXCL9* and *IDO1 n *=* *6), gt3‐infected patients (*n *=* *9) and controls (*n *=* *6, except *CXCL9* and *IDO1 n *=* *4). * denotes *P*‐value <0.05, ** denotes *P*‐value <0.01, and *** denotes *P*‐value <0.001.

To explore viral genotype‐specific changes in liver gene expression, five of each of the gt1‐ and gt3‐infected specimens were selected for high‐throughput sequence analysis, on the basis of low fibrosis scores (METAVIR fibrosis score ≤1) and RIN values >6.5 (Table S2). From among the available uninfected biopsies, 4 samples were selected such that the control group included conditions associated with liver disease as well as a normal liver sample (Table S2). This approach was adopted to bias the resultant hits towards those regulated exclusively by HCV rather than liver inflammation in general, although this possibly reduced the number of hits that may have been obtained if solely healthy liver samples were utilised as controls. RNA‐Seq generated ~17–25 million reads per sample, of which 82–87% uniquely mapped to the human genome (Table S2). All filtered sequence libraries had high quality, with >90% of reads having a total mean Phred quality score ≥32 and a mean per base Phred quality score of >34 (Fig. 1).

We identified a total of 348 and 565 genes that were differentially regulated (upregulated or downregulated with ∆FPKM(log2) ≥1.0 and q‐value <0.05 as detailed in the supplementary data and summarised in Figure 1) in the gt1‐ and gt3‐infected samples, respectively, compared to controls. A total of 196 genes were common to both gene sets (56% of the total differentially regulated genes in the gt1 samples and 35% of the total differentially regulated genes in the gt3 samples).

To directly explore viral genotype‐specific transcriptional profiles, we analysed the sequencing data to identify genes differentially regulated between the gt1‐ and gt3‐infected biopsies. A total of 149 genes were differentially regulated between the gt1‐ and gt3‐infected samples with ∆FPKM(log2) ≥1.0 and q‐value <0.05 (64 upregulated in gt3, 85 upregulated in gt1; Figure S2 and supplementary data). The finding that a significant number of genes are differentially regulated between the viral genotypes, together with the low level of overlap between the transcriptional profiles generated comparing either gt1‐ or gt3‐infected samples to controls, highlights that viral genotype has a large influence on the regulation of host genes during chronic HCV infection.

To validate the RNA‐Seq results, several differentially regulated genes were examined by Q‐PCR using all available 24 liver RNA samples. The selected targets included genes that were differentially expressed in both gt1‐ and gt3‐infected samples versus controls (*CXCL9, IFIT1, ISG15* and *RSAD2*), as well as genes that were differentially regulated only in gt1 infection (*USP18*) or gt3 infection (*IDO1*). The Q‐PCR data corresponded closely to the sequencing read counts confirming the RNA‐Seq results (Fig. 1B).

### Enriched canonical pathways differ between HCV gt1 and gt3 infections

To explore potential biological processes underlying the observed transcriptional changes, the differentially regulated genes identified were analysed for pathway enrichment. The top enriched canonical pathways in the gt1‐ and gt3‐infected liver biopsies compared to the controls differed markedly, with only three shared pathways in the top 10 enriched pathways, paralleling the small proportion of differentially regulated genes common to both comparisons (Table S3). While innate immune pathways were evident in the gt1‐infected liver biopsies (e.g. activation of IRF by cytosolic pattern recognition receptors and interferon signalling), pathways relating to adaptive immune responses were more apparent in the gt3‐infected liver biopsies (e.g. altered T‐ and B‐cell signalling in rheumatoid arthritis, calcium‐induced T‐lymphocyte apoptosis, iCOS‐iCOSL signalling in T‐helper cells, B‐cell development and CD28 signalling in T‐helper cells). Despite the clinical association of gt3 infection with increased prevalence of liver steatosis and decreased peripheral cholesterol levels 7, no enrichment of lipid or cholesterol metabolic pathways occurred in the gt3‐infected samples.

Pathway analysis was also performed on the 149 genes identified as differentially regulated between gt1‐ and gt3‐infected liver biopsies. Among the top enriched canonical pathways, the antigen presentation pathway had the highest enrichment in gt3‐infected samples, with B‐cell development, allograft rejection signalling and altered T‐ and B‐cell signalling in rheumatoid arthritis, also enriched in the gt3‐infected patients (Table 2). The differentially regulated genes within these pathways include HLA genes involved in presentation of antigen to T‐helper cells (HLA‐DQB2, HLA‐DRB1, HLA‐DMA, HLA‐DRA) as well as CD74, also known as the HLA invariant chain, which acts as a chaperone to regulate antigen presentation. Conversely, the interferon signalling pathway was enriched in the gt1‐infected liver biopsies (Table 2), due to the elevated expression of a number of known ISGs, such as OAS1, MX1 and IFIT1.

**Table 2:** Top 5 canonical pathways between gt1‐ and gt3‐infected liver biopsies

Gt1 vs Gt3	*P*‐value (−log)	Ratio^a^	Up‐ or Downregulated in Gt1^b^
Antigen Presentation Pathway	5.48	0.135	Down
B‐Cell Development	2.91	0.088	Down
Interferon Signalling	2.83	0.083	Up
Allograft Rejection Signalling	2.68	0.047	Down
Altered T‐Cell and B‐Cell Signalling in Rheumatoid Arthritis	2.64	0.046	Down

a Ratio of list genes found per pathway over the total number of genes in that pathway.

b Based on the up‐ or downregulation of individual differentially regulated genes belonging to the canonical pathway.

### Type II IFN expression is elevated in HCV gt3 infection

The RNA‐Seq data indicated that both gt1 and gt3 induced transcription of a number of ISGs within the liver, and this ISG induction was particularly evident in gt1 infection. To explore potential upstream regulators of the ISG response, we profiled transcription levels of type I, type II and type III IFNs, including *IFNA2*,* IFNB1*,* IFNL2* and *IFNG*.

**Figure 2:**
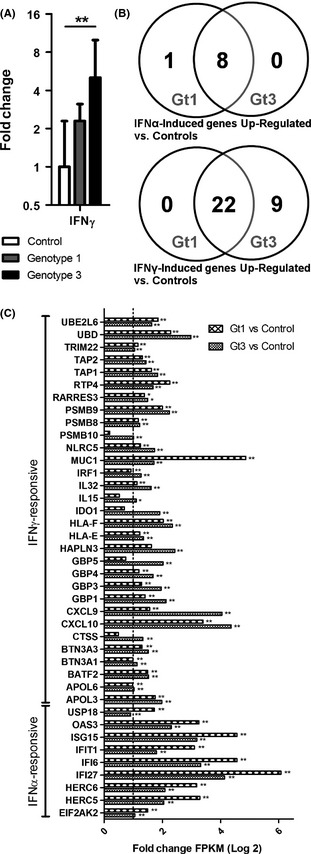
Type II IFN transcription and downstream gene induction are elevated in HCV gt3 infection. (A) *IFNG* mRNA levels (geometric mean with 95% confidence interval) in gt1‐infected patients (*n *=* *9), gt3‐infected patients (*n *=* *9) and controls (*n *=* *6). ** denotes *P*‐value <0.01. (B) Preferentially IFN‐α‐ and IFN‐γ‐responsive genes identified in HuH‐7 and HepaRG cells and upregulated in liver biopsies from either gt1‐ (*n *=* *5) or gt3‐infected patients (*n *=* *5), versus controls (*n *=* *4). (C) Fold change of preferentially IFN‐α‐ and IFN‐γ‐regulated genes in gt1‐ and gt3‐infected liver biopsies versus control liver biopsies. The dotted line represents the ∆FPKM(log2) ≥1.0 cut‐off used to select differentially regulated genes. * denotes q‐value <0.05, and ** denotes q‐value <0.01.

We detected expression of *IFNA2*,* IFNB1* and *IFNL2* in fewer than half of the biopsy samples and could not reliably quantify expression levels (data not shown). This low level of type I and type III IFN expression was also observed in the RNA‐Seq data (data not shown), indicating that elevated type I and type III IFN expression is unlikely to directly account for the transcriptional changes observed in gt1‐infected liver biopsies. Likewise, the role of type I, type II and type III IFN receptors could also be excluded as they were stably expressed across all biological groups (data not shown). Of the 4 tested IFN genes, only *IFNG* was expressed at a level that allowed reliable detection in liver tissues. *IFNG* expression was significantly higher in gt3‐infected patients but did not differ significantly in gt1 infection compared to the control group (Fig. 2A). Several genes identified within the gt3‐specific enriched pathways are induced by IFN‐γ, suggesting that the observed increase in *IFNG* gene expression may contribute to upstream regulation of these gt3‐specific cellular pathways.

### Identification of IFN‐α‐ and IFN‐γ‐regulated genes in human hepatocyte‐derived cell lines

As the transcriptional changes induced by different types of IFN overlap significantly 19, 20, it is difficult to classify ISGs as exclusively responsive to a particular type of IFN. Many ISGs do however show a preferential response to either type I or type II IFN. To identify particular ISGs which are preferentially induced by either IFN‐α or IFN‐γ in hepatocytes, we tested the effect of stimulation by both IFNs on 2 hepatocyte‐derived cell lines, HuH‐7 and HepaRG cells. By comparing the ratio of the IFN‐α/IFN‐γ fold change values in HuH‐7 and HepaRG cells 24 hours following stimulation, we identified 14 preferentially IFN‐α‐responsive genes and 74 preferentially IFN‐γ‐responsive genes, 3 of which were consistently downregulated by IFN‐γ (Table S4). Comparing the *in vitro* and *in vivo* results, the gt1‐infected liver biopsies had a greater upregulation of 9 of the 14 identified preferentially IFN‐α‐responsive genes, while gt3‐infected liver biopsies had a greater upregulation of 18 of the 22 identified preferentially IFN‐γ‐responsive genes (Fig. 2B and C). These results support a role for IFN‐γ in driving gene transcription in the context of gt3 infection, while HCV gt1 infection is associated with a type I IFN‐like transcriptional signature.

### The influence of *IFNL4* polymorphisms on ISG expression is evident in gt1 infection but not gt3 infection

Previous studies have linked polymorphisms rs12979860 and rs8099917 located near *IFNL3* to HCV susceptibility 21 and response to IFN‐α‐based therapy 22, 23. While the biological mechanisms underlying this association are debated, elevated induction of ISGs is observed in individuals carrying the unfavourable polymorphisms 24. More recent genetic analysis has revealed another polymorphism (rs368234815; previously designated ss469415590) in exon 1 of *IFNL4*, which is in high linkage disequilibrium with rs12979860 and, indeed, is a better marker for HCV clearance in certain ethnic populations 18. The expression of IFNL4 in tissue culture cells upregulates a number of ISGs. However, the rs368234815 TT variant results in a frameshift which disrupts the expression of IFNL4.

**Figure 3:**
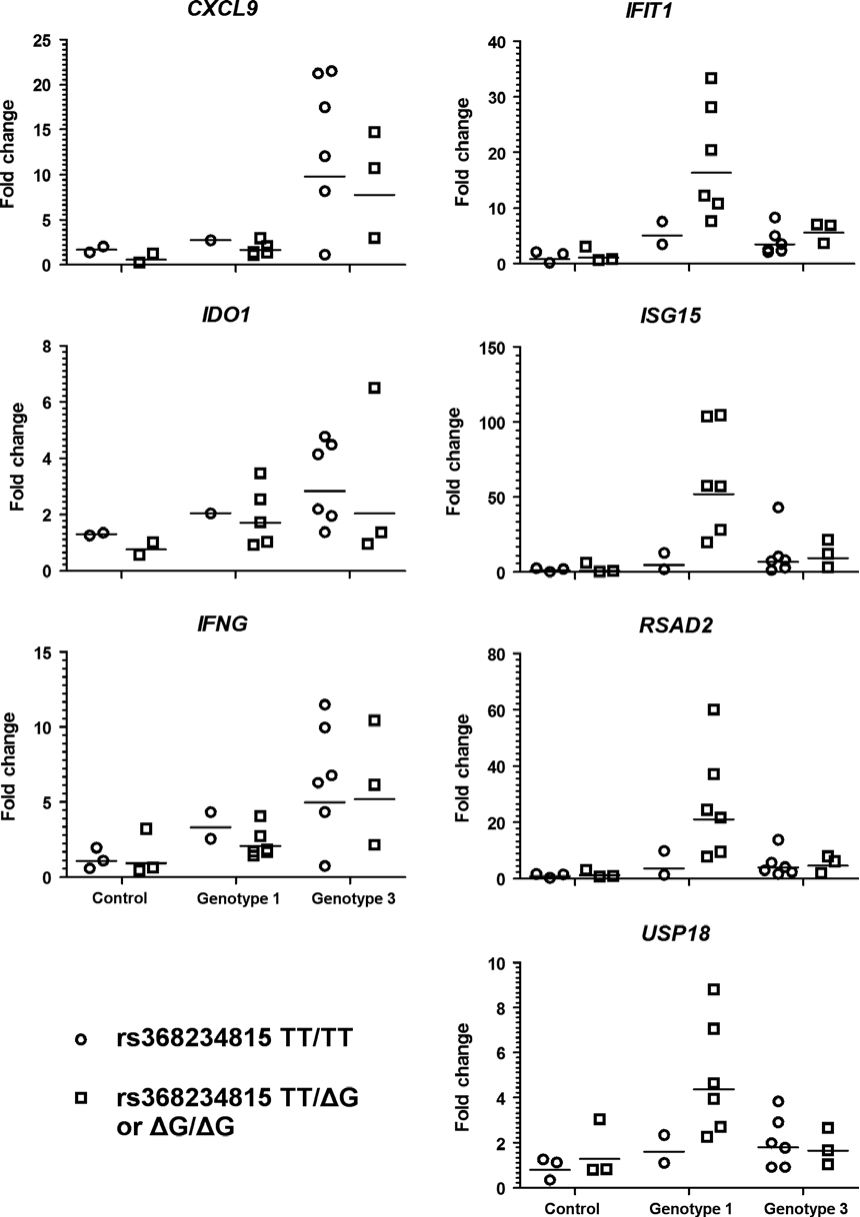
ISG mRNA transcription in gt3‐infected liver biopsies is not influenced by IFNL4 genotype. Dot plots of Q‐PCR data segregated by *IFNL4* (rs368234815) genotype (with geometric mean denoted by a horizontal line). Samples with the TT/TT genotype are plotted as circles, while samples with the ΔG/TT or ΔG/ΔG are plotted as squares. Fold changes values are calculated relative to the mean of the control group. For the control group, *n *=* *3 for both the TT/TT and ΔG/TT or ΔG/ΔG genotypes (except for CXCL9 and IDO9 where *n *=* *2 for both genotypes). For the gt1‐infected group, *n *=* *2 for the TT/TT genotype and *n *=* *6 for the ΔG/TT or ΔG/ΔG genotypes (except for CXCL9 and IDO9 where *n *=* *1 and *n *=* *5 respectively). For the gt3‐infected group, *n *=* *6 for the TT/TT genotype and *n *=* *3 for the ΔG/TT or ΔG/ΔG genotypes.

To determine whether the observed differences in gene expression were related to not only viral genotype but also host genetic background, we performed *IFNL4* genotyping (rs368234815) on all but one of the samples used in our study (as detailed in Table 1). As shown in Figure 3, the presence of the ΔG variant, which would lead to expression of IFNL4, correlated with higher expression levels of ISGs such as *IFIT1*,* ISG15*,* RSAD2* and *USP18*, within the gt1‐infected group. Due to the low number of samples with TT/TT genotype, statistical analysis could not be performed within the gt1‐infected group; however, our results parallel previous studies of gt1‐infected individuals 24. In stark contrast, the expression of genes specifically induced in gt3‐infected biopsies, such as *CXCL9*,* IDO1* and *IFNG*, was not significantly different regardless of *IFNL4* genotype (Fig. 3).

## Discussion

Using high‐throughput transcriptome sequencing, we identified distinct hepatic transcriptional profiles associated with different viral genotypes within HCV‐infected liver biopsies. Gt1 infection correlated with elevated type I IFN‐like ISG transcription, while gt3 infection was associated with higher transcription of genes involved in cell‐mediated immune responses and IFN‐γ signalling. While previous studies have documented the induction of type I IFN‐inducible ISGs and antiviral cellular pathways during chronic HCV infection, our results highlight that elevated ISG transcription is dependent on viral genotype. Hence, in contrast to gt1, gt3 infection is associated with the enrichment of pathways relating to adaptive immune responses and a weaker induction of ISGs. Despite the clinical association of gt3 infection with liver steatosis, we failed to detect any transcriptional differences in lipid metabolic pathways in these patients with early stages of liver disease. A recent metabolic study has identified a gt3‐specific decrease in the serum levels of cholesterol metabolites 25, suggesting that changes associated with gt3 infection and steatosis may only be evident at a post‐transcriptional level.

The pathways identified as regulated by HCV in previous studies vary considerably. Such variability undoubtedly emerges from differences in the physiological state of liver samples at time of collection, the extent of liver disease and distinct host genetic backgrounds. We have tried to minimise such variability by excluding liver samples with evidence of cirrhosis and using both normal liver samples, as well as samples from uninfected patients with conditions associated with hepatic disease, as controls. This approach has the impact of lowering the number of genes identified due to generic pathological processes within the liver, enabling a greater likelihood of determining host genes that are specifically regulated by HCV. Consequently, we believe that the resultant RNA‐Seq data provide greater certainty that the up‐ and downregulation of cellular genes is virus mediated.

A central question arising from our study is why gt1 and gt3 mediate such different hepatic transcriptional responses. While we observed a general induction of ISGs by both genotypes compared to controls, the panel of induced ISGs was not identical. Whereas there is persistent induction of ISGs in gt1 infection, gt3 promotes differential expression of a larger set of genes, including factors stimulated by IFN‐γ. We did not observe alterations in either type I or type III IFN transcription in the infected or uninfected biopsies. However, IFN‐γ mRNA was expressed at significantly higher levels in gt3 infection and is associated with elevated expression of type II IFN‐regulated genes. Elevated expression of type I IFNs is known to regulate responses to type II IFNs 26, 27, via downregulation of the IFN‐γ receptor 28. These exaggerated type I IFN responses can drive immunopathology and antagonise protective IFN‐γ responses during bacterial infections 29. In the context of HCV infection, exaggerated type I IFN‐like responses may subvert the development of protective cell‐mediated immune responses and suppress the expression of antiviral mediators that are driven by IFN‐γ signalling 20. The reduced expression of HLA genes in gt1 infection compared to gt3 potentially impacts on antigen presentation within the liver and may lead to the development of defective cellular immune responses during gt1 infection.

From the perspective of treatment, high levels of ISGs are associated with poor response to therapy 15, 24, 30. Basal activation of the type I IFN signalling pathway induces a state of refractoriness to further stimulation 31, most likely through the action of ISGs that act as negative regulators of IFN‐α signalling, such as the SOCS proteins and USP18 32, 33, 34. Elevated ISG induction in the liver is associated with *IFNL3* polymorphisms 24, although this association is not apparent within peripheral blood 35. The apparent discrepancy between peripheral blood and liver suggests that the effect of IFN‐λ polymorphisms is strongest at the local site of HCV infection within the liver. These findings highlight that both viral and host factors influence ISG induction and the relative influence varies between local sites of infection and the periphery.

While the mechanisms underlying the relationship between IFN‐λ polymorphism, liver ISG induction and treatment non‐response are still unclear, recent studies have suggested that the unfavourable polymorphisms are associated with increased IFN‐λ receptor 1 expression 36 and enhanced AU‐rich element‐mediated decay of *IFNL3* mRNA 37. No differences were detected in the expression levels of any of the IFN‐λ mRNAs, nor in the mRNA expression of the unique *IFNLR1* receptor subunit in our samples. However, the induction of ISGs in gt1‐infected liver biopsies did correlate with the presence of the ΔG allele in *IFNL4*. By contrast, there was no evidence for a link between *IFNL4* genotype and changes in gene expression in gt3 infection. These data provide a rationale for the reduced efficacy of *IFNL* SNPs in predicting gt3 treatment outcomes 38.

Our data demonstrate that gt1 infection drives a highly skewed type I IFN‐like response, which is apparently influenced by host *IFNL4* genotype. Conversely, gt3 induces upregulation of IFN‐γ and IFN‐γ‐responsive ISGs, independent of host *IFNL4* genotype. These transcriptional differences highlight fundamental differences in host–pathogen interactions that exist between different HCV genotypes and provide a platform for further research into the impact of viral genotype on host responses. Future studies that focus on changes in liver gene expression elicited by other genotypes will be important to understand how the natural history of infection is influenced by viral variation. Such knowledge will undoubtedly be vital to develop strategies for predicting outcomes of therapy and combating the different HCV genotypes circulating within human populations.

## Supporting information


**Table S1.** Gene selection for Q‐PCR assays.
**Table S2.** Liver biopsy RNA‐Seq total reads and % mapped to human genes.
**Table S3.** Top 10 canonical pathways in gt1‐ and gt3‐infected liver biopsies versus controls.
**Table S4.** Genes preferentially responsive to either IFN‐α or IFN‐γ in HepaRG and HuH‐7 cell lines.
**Fig. S1.** Filtered RNA‐Seq sequencing library quality scores.
**Fig. S2.** Viral genotype‐specific transcriptional changes within the liver.
**Data S2.** Methods.

**Data S1.** RNA‐Seq Significant Genes.
