# Supplementary material for: Viral genotype correlates with distinct liver gene transcription signatures in chronic hepatitis C virus infection
Source: Liver Int. 2015 Apr 7;35(10):2256–64. doi: 10.1111/liv.12830 (PMC4949513; doi:10.1111/liv.12830)
Supplement: Supplementary file 1 — Table S1. Gene selection for Q‐PCR assays. Table S2. Liver biopsy RNA‐Seq total reads and % mapped to human genes. Table S3. Top 10 canonical pathways in gt1‐ and gt3‐infected liver biopsies versus controls. Table S4. Genes preferentially responsive to either IFN‐α or IFN‐γ in HepaRG and HuH‐7 cell lines. Fig. S1. Filtered RNA‐Seq sequencing library quality scores. Fig. S2. Viral genotype‐specific transcriptional changes within the liver. Data S2. Methods. [file LIV-35-2256-s001.doc]

**Supplementary Methods**

The RNA-seq analysis was performed using Tuxedo protocol [1] as follow: First, in order to reduce the mapping time for each sample a transcriptome index was created using TopHat v2.0 [2] based on the build GRCh 37 (hg19) downloaded from iGenomes with the following options: -G UCSC_hg19_genes.gtf -p 8 and --transcriptome-index=know. The reads for each library were mapped using TopHat against the hg19 with the default options except for: -p 8 --solexa-quals --transcriptome-index=know. Gene expression was calculated using Cufflinks v2.0 [3] with parameter set at: --GTF-guide UCSC_hg19_genes.gtf -p 8 --frag-bias-correct --multi-read-correct. The resulting Cufflinks assemblies of all samples were combined using Cuffmerge [1] (-g UCSC_hg19_genes.gtf and –s UCSC_hg19_genome.fa) and then Cuffdiff2 [3] was used to identify differentially expressed genes with parameters set at: -u merged.gtf –b genome.fa. Significant gene expression was considered when the observed log2-fold-change of reads (FPKM) was ≥ 1.0 and FDR/q-value < 0.05 between comparisons. In order to visualize tabular data, we used Circos Table Viewer v0.63-9 [4].

1. Trapnell, C., et al., *Differential gene and transcript expression analysis of RNA-seq experiments with TopHat and Cufflinks.* Nat Protoc, 2012. **7**(3): p. 562-78.

2. Kim, D., et al., *TopHat2: accurate alignment of transcriptomes in the presence of insertions, deletions and gene fusions.* Genome Biol, 2013. **14**(4): p. R36.

3. Trapnell, C., et al., *Differential analysis of gene regulation at transcript resolution with RNA-seq.* Nat Biotechnol, 2013. **31**(1): p. 46-53.

4. Krzywinski, M., et al., *Circos: an information aesthetic for comparative genomics.* Genome Res, 2009. **19**(9): p. 1639-45.

**Supplementary Table 1: Gene selection for Q-PCR assays**

| HGNC Gene Symbol | Gene Name | Applied Biosystems TaqMan Assay |
| --- | --- | --- |
| *GAPDH* | Glyceraldehyde-3-phosphate dehydrogenase | 4326317E |
| *HRPT1* | Hypoxanthine phosphoribosyltransferase 1 | 4333768F |
| *CXCL9* | Chemokine (C-X-C Motif) Ligand 9 | Hs00171065_m1 |
| *IDO1* | Indoleamine 2,3-dioxygenase 1 | Hs00984148_m1 |
| *IFIT1* | Interferon-induced protein with tetratricopeptide repeats 1 | Hs01911452_s1 |
| *ISG15* | ISG15 ubiquitin-like modifier | Hs01921425_s1 |
| *RSAD2* | Radical S-adenosyl methionine domain containing 2 | Hs00369813_m1 |
| *TNFSF10* | Tumour necrosis factor (ligand) superfamily, member 10 | Hs00921974_m1 |
| *USP18* | Ubiquitin specific peptidase 18 | Hs00276441_m1 |
| *XAF1* | XIAP associated factor 1 | Hs01550142_m1 |
| *IFNA2* | Interferon, alpha 2 | Hs00265051_s1 |
| *IFNB1* | Interferon, beta 1 | Hs01077958_s1 |
| *IFNG* | Interferon, gamma | Hs00989291_m1 |
| *IFNL2* | Interferon, lambda 2 | Hs00820125_g1 |

**Supplementary Table 2**: Liver biopsy RNA-Seq total reads and % mapped to human genes

| **Group** | **Sample ID** | **Diagnosis** | **Gender** | ***IFNL* genotype (rs368234815)** | **RIN*** | **Total reads** | **% Uniquely mapped reads** |
| --- | --- | --- | --- | --- | --- | --- | --- |
| Genotype 1 | 1 | HCV | M | *TT/ΔG* | 7.1 | 19390849 | 83.62 |
| Genotype 1 | 2 | HCV | M | *TT/TT* | 7.8 | 22502516 | 82.36 |
| Genotype 1 | 3 | HCV | M | *TT/ΔG* | 8.3 | 24691491 | 82.67 |
| Genotype 1 | 4 | HCV | M | *TT/ΔG* | 7.9 | 21115332 | 84.90 |
| Genotype 1 | 5 | HCV | F | *TT/ΔG* | 8 | 21517212 | 87.11 |
| Genotype 3 | 6 | HCV | M | *TT/TT* | 6.6 | 20792197 | 85.91 |
| Genotype 3 | 7 | HCV | M | *TT/ΔG* | 8 | 21058132 | 86.43 |
| Genotype 3 | 8 | HCV | M | *TT/TT* | 7.2 | 19902380 | 86.13 |
| Genotype 3 | 9 | HCV | M | *TT/TT* | 6.6 | 22370300 | 86.65 |
| Genotype 3 | 10 | HCV | M | *TT/ΔG* | 7.7 | 22213190 | 87.00 |
| Control | 11 | Metastatic colorectal adenocarcinoma | - | *TT/ΔG* | 8.8 | 18870257 | 84.04 |
| Control | 12 | Genetic haemochromatosis | F | *TT/TT* | 9.1 | 24037648 | 86.17 |
| Control | 13 | Non-alcoholic steatohepatitis | M | *TT/ΔG* | 8.8 | 16921472 | 85.68 |
| Control | 14 | Psoriasis | M | *TT/TT* | 8.7 | 20731884 | 84.87 |

* RIN = RNA integrity number

**Supplementary Table 3: Top 10 canonical pathways in gt1- and gt3-infected liver biopsies versus controls**

| **Gt1 vs Controls** | ***P*-value (-log)** | **Ratio*** | **Gt3 vs Controls** | ***P*-value (-log)** | **Ratio*** |
| --- | --- | --- | --- | --- | --- |
| Antigen Presentation Pathway | 13.00 | 0.32 | Antigen Presentation Pathway | 26.10 | 0.60 |
| Interferon Signaling | 10.20 | 0.28 | Altered T Cell and B Cell Signaling in Rheumatoid Arthritis | 13.80 | 0.23 |
| Type I Diabetes Mellitus Signaling | 7.13 | 0.11 | Calcium-induced T Lymphocyte Apoptosis | 13.00 | 0.27 |
| Activation of IRF by Cytosolic Pattern Recognition Receptors | 6.44 | 0.14 | iCOS-iCOSL Signaling in T Helper Cells | 13.00 | 0.19 |
| OX40 Signaling Pathway | 6.16 | 0.11 | OX40 Signaling Pathway | 12.60 | 0.21 |
| Cdc42 Signaling | 5.96 | 0.08 | B Cell Development | 12.40 | 0.38 |
| Graft-versus-Host Disease Signaling | 5.24 | 0.15 | CD28 Signaling in T Helper Cells | 12.20 | 0.18 |
| Autoimmune Thyroid Disease Signaling | 5.17 | 0.14 | Primary Immunodeficiency Signaling | 12.20 | 0.29 |
| Dendritic Cell Maturation | 4.88 | 0.07 | Dendritic Cell Maturation | 12.00 | 0.14 |
| IL-17A Signaling in Gastric Cells | 4.57 | 0.20 | Allograft Rejection Signaling | 11.80 | 0.21 |

* Ratio of list genes found per pathway over the total number of genes in that pathway

**Supplementary Table 4: Genes preferentially** responsive to either IFNα or IFNγ in HepaRG and HuH-7 cell lines

| **Gene Symbol** | **Fold Change HepaRG IFNα-treated** | **Fold Change HuH-7 IFNα-treated** | **Fold Change HepaRG IFNγ-treated** | **Fold Change HuH-7 IFNγ-treated** | **IFNα or IFNγ Responsive** |
| --- | --- | --- | --- | --- | --- |
| *DDX58* | 47.6 | 22.7 | 23.7 | 3.5 | Alpha |
| *DHRS2* | 2.0 | 4.0 | 0.7 | 1.2 | Alpha |
| *EIF2AK2* | 3.6 | 7.9 | 1.8 | 2.3 | Alpha |
| *HERC5* | 9.2 | 9.9 | 1.5 | 0.8 | Alpha |
| *HERC6* | 22.4 | 135.7 | 10.1 | 27.4 | Alpha |
| *IFI27* | 190.1 | 72.0 | 35.2 | 7.8 | Alpha |
| *IFI44* | 43.7 | 200.5 | 14.0 | 17.2 | Alpha |
| *IFI6* | 80.5 | 527.6 | 13.5 | 24.0 | Alpha |
| *IFIT1* | 14.9 | 324.9 | 2.7 | 6.9 | Alpha |
| *ISG15* | 87.5 | 68.9 | 23.0 | 12.9 | Alpha |
| *OAS3* | 8.7 | 53.9 | 4.3 | 10.5 | Alpha |
| *PPM1K* | 2.0 | 2.1 | 0.9 | 0.9 | Alpha |
| *TRGV7* | 9.0 | 9.2 | 1.3 | 0.4 | Alpha |
| *USP18* | 6.0 | 7.6 | 1.6 | 1.8 | Alpha |
| *ACY3* | 1.1 | 2.5 | 5.8 | 6.5 | Gamma |
| *APOL1* | 1.3 | 2.3 | 14.9 | 8.1 | Gamma |
| *APOL3* | 1.3 | 0.9 | 18.3 | 3.6 | Gamma |
| *APOL4* | 2.7 | 2.9 | 16.4 | 6.2 | Gamma |
| *APOL6* | 2.8 | 4.5 | 15.4 | 18.1 | Gamma |
| *ATP1A2* | 0.9 | 2.3 | 0.2 | 0.5 | Gamma |
| *BATF2* | 29.4 | 11.4 | 455.9 | 50.1 | Gamma |
| *BTN3A1* | 1.8 | 1.8 | 5.8 | 3.6 | Gamma |
| *BTN3A3* | 1.8 | 2.8 | 4.9 | 7.7 | Gamma |
| *C14orf132* | 0.8 | 1.0 | 3.3 | 2.3 | Gamma |
| *C1R* | 4.3 | 5.2 | 22.0 | 29.0 | Gamma |
| *C1S* | 2.3 | 2.9 | 7.4 | 11.2 | Gamma |
| *C2* | 0.9 | 2.2 | 7.6 | 7.4 | Gamma |
| *C4B* | 1.3 | 5.6 | 136.0 | 68.6 | Gamma |
| *C5orf56* | 3.4 | 3.5 | 19.0 | 10.2 | Gamma |
| *CA11* | 0.9 | 1.0 | 2.4 | 3.0 | Gamma |
| *CAND2* | 0.8 | 0.8 | 87.3 | 4.3 | Gamma |
| *CD274* | 1.9 | 3.8 | 52.5 | 49.7 | Gamma |
| *CTSS* | 2.2 | 6.6 | 175.0 | 16.0 | Gamma |
| *CXCL10* | 1.3 | 19.2 | 110.2 | 597.2 | Gamma |
| *CXCL11* | 2.5 | 2.1 | 17.9 | 25.8 | Gamma |
| *CXCL9* | 0.9 | 12.2 | 284.0 | 1024.8 | Gamma |
| *CYP1B1* | 1.0 | 1.3 | 2.0 | 2.7 | Gamma |
| *CYP21A2* | 1.0 | 1.2 | 3.9 | 4.2 | Gamma |
| *ERAP2* | 2.1 | 4.0 | 8.8 | 10.8 | Gamma |
| *FAM20A* | 1.2 | 1.3 | 74.8 | 4.0 | Gamma |
| *FBXO6* | 3.0 | 2.6 | 16.0 | 6.3 | Gamma |
| *GBP1* | 9.4 | 27.1 | 284.9 | 836.4 | Gamma |
| *GBP2* | 0.9 | 1.5 | 14.0 | 7.8 | Gamma |
| *GBP3* | 4.0 | 14.2 | 43.8 | 67.8 | Gamma |
| *GBP4* | 1.1 | 1.0 | 32.5 | 14.5 | Gamma |
| *GBP5* | 1.4 | 1.4 | 62.4 | 880.4 | Gamma |
| *GPR15* | 1.6 | 302.2 | 0.3 | 0.2 | Gamma |
| *HAPLN3* | 1.1 | 5.0 | 14.7 | 26.6 | Gamma |
| *HK1* | 1.0 | 1.2 | 2.3 | 3.0 | Gamma |
| *HLA-E* | 2.6 | 5.9 | 6.8 | 28.5 | Gamma |
| *HLA-F* | 2.9 | 5.2 | 9.6 | 13.2 | Gamma |
| *ICAM1* | 1.1 | 2.4 | 7.4 | 6.6 | Gamma |
| *IDO1* | 2.0 | 0.9 | 2367.8 | 38.8 | Gamma |
| *IGFLR1* | 1.1 | 1.3 | 6.5 | 2.7 | Gamma |
| *IL15* | 1.9 | 3.2 | 12.8 | 7.9 | Gamma |
| *IL15RA* | 1.5 | 2.8 | 6.5 | 35.4 | Gamma |
| *IL18BP* | 0.9 | 2.5 | 212.2 | 24.5 | Gamma |
| *IL32* | 1.1 | 2.4 | 21.9 | 4.9 | Gamma |
| *IRF1* | 1.6 | 3.4 | 64.9 | 34.6 | Gamma |
| *KLHDC7B* | 5.2 | 9.7 | 17.9 | 21.4 | Gamma |
| *KRT4* | 0.8 | 1.0 | 0.3 | 0.5 | Gamma |
| *LOC100131733* | 1.4 | 4.7 | 194.8 | 141.2 | Gamma |
| *MMP25* | 0.9 | 1.0 | 2.2 | 2.3 | Gamma |
| *MUC1* | 1.2 | 2.4 | 3.4 | 6.6 | Gamma |
| *NLRC5* | 1.7 | 13.6 | 6.6 | 236.7 | Gamma |
| *NNMT* | 1.1 | 1.9 | 3.2 | 146.2 | Gamma |
| *PRRG4* | 1.0 | 1.0 | 2.1 | 2.5 | Gamma |
| *PSMB10* | 1.4 | 1.3 | 11.2 | 3.4 | Gamma |
| *PSMB8* | 2.4 | 36.0 | 12.8 | 245.9 | Gamma |
| *PSMB9* | 4.7 | 10.8 | 47.6 | 132.7 | Gamma |
| *RARRES3* | 6.0 | 19.0 | 71.1 | 246.8 | Gamma |
| *RTP4* | 1.5 | 26.1 | 154.3 | 85.4 | Gamma |
| *SERPING1* | 2.9 | 2.1 | 343.5 | 6.6 | Gamma |
| *SLC22A2* | 2.2 | 1.0 | 22.4 | 2.2 | Gamma |
| *SOCS3* | 1.2 | 2.0 | 5.3 | 6.6 | Gamma |
| *SQRDL* | 1.0 | 1.5 | 2.5 | 3.8 | Gamma |
| *TAP1* | 3.0 | 154.8 | 25.0 | 2198.8 | Gamma |
| *TAP2* | 1.5 | 2.3 | 4.9 | 8.7 | Gamma |
| *TAPBPL* | 2.0 | 3.0 | 16.0 | 6.9 | Gamma |
| *TGM2* | 1.1 | 1.1 | 2.8 | 2.9 | Gamma |
| *TNFRSF14* | 1.2 | 4.5 | 15.9 | 60.5 | Gamma |
| *TNFSF14* | 0.9 | 1.9 | 6.5 | 16.4 | Gamma |
| *TRIM15* | 1.0 | 1.4 | 2.3 | 3.4 | Gamma |
| *TRIM22* | 11.0 | 7.3 | 113.4 | 16.4 | Gamma |
| *UBD* | 1.1 | 6.0 | 102.9 | 27.9 | Gamma |
| *UBE2L6* | 8.7 | 17.0 | 136.6 | 53.2 | Gamma |
| *WARS* | 1.1 | 1.0 | 20.1 | 3.4 | Gamma |

To minimise cell line-specific effects, genes were classified as either preferentially IFNα- or IFNγ-responsive if: i) the fold change values were >2 or <0.5 in both HuH-7 and HepaRG cells, and ii) the ratio of the IFNα/IFNγ fold change values was >2 or <0.5 in both cell lines.

**Supplementary Figure 1: Filtered RNA-Seq sequencing library quality scores**

**
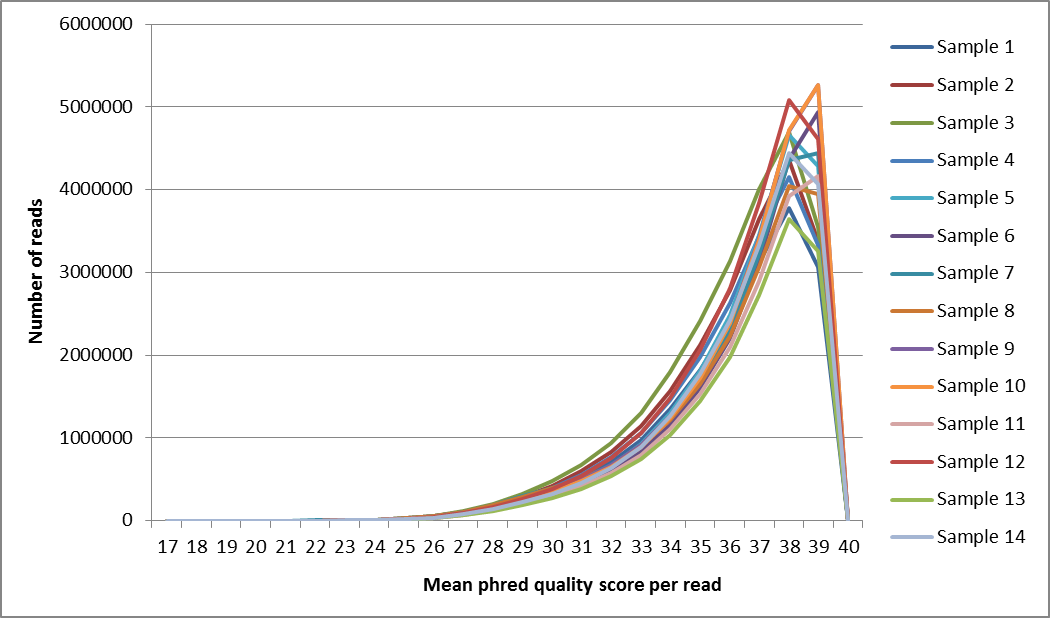
**

**
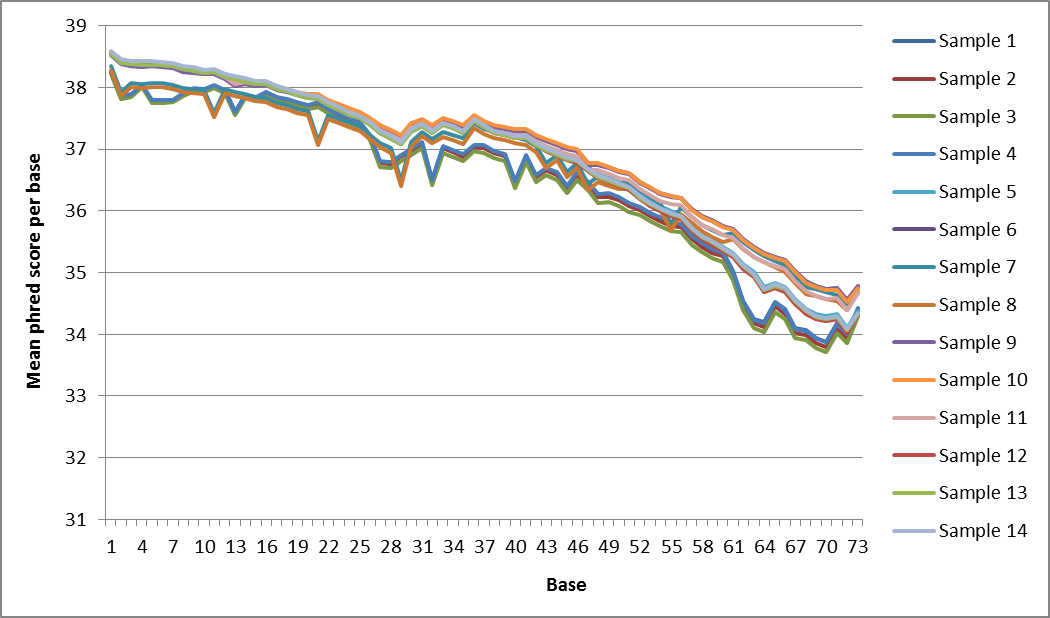
**

**Supplementary Figure 2: Viral genotype-specific transcriptional changes within the liver.** Circos plots illustrating the top 100 differentially expressed genes sorted on expression levels in liver biopsies of gt1- and gt3-infected patients (*n*=5 each). The thickness of link between the sample name and the gene name represents the average of the gene expression (FPKM value).

**
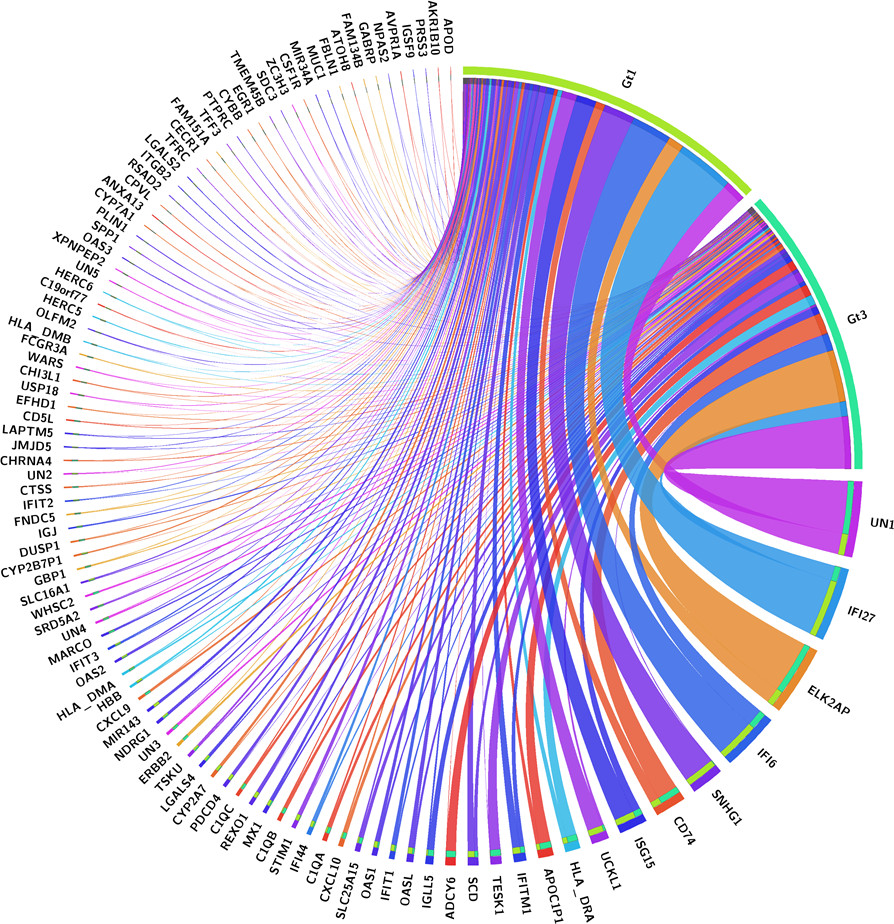
**
